# Supplementary material for: Macrophage Polarization Status Impacts Nanoceria Cellular Distribution but Not Its Biotransformation or Ferritin Effects
Source: Nanomaterials (Basel). 2023 Aug 10;13(16):2298. doi: 10.3390/nano13162298 (PMC10459093; doi:10.3390/nano13162298)
Supplement: Supplementary file 1 [file nanomaterials-13-02298-s001.zip › nanomaterials-2510334-supplementary.pdf]

nanomaterials

Supporting Information:

Macrophage Polarization Status Impacts Nanoceria Cellular Distribution but Not Its Biotransformation or Ferritin Effects

Uschi M. Graham [1], Alan K. Dozier [2], David J. Feola [3], Michael T. Tseng [4,†] and Robert A. Yokel [1,*]

[1] Pharmaceutical Sciences Department, College of Pharmacy, University of Kentucky, Lexington, KY 40536-0596, USA; graham@topasol.com
[2] National Institute of Occupational Safety and Health (NIOSH), Cincinnati, OH 45213-2515, USA; xlh5@cdc.gov
[3] Pharmacy Practice and Science Department, College of Pharmacy, University of Kentucky, Lexington, KY 40536-0596, USA; david.feola@uky.edu
[4] Anatomical Sciences and Neurobiology, University of Louisville, Louisville, KY 40202, USA
* Correspondence: ryokel@uky.edu; Tel.: +1-859-257-4855
† Deceased 17 December 2019.

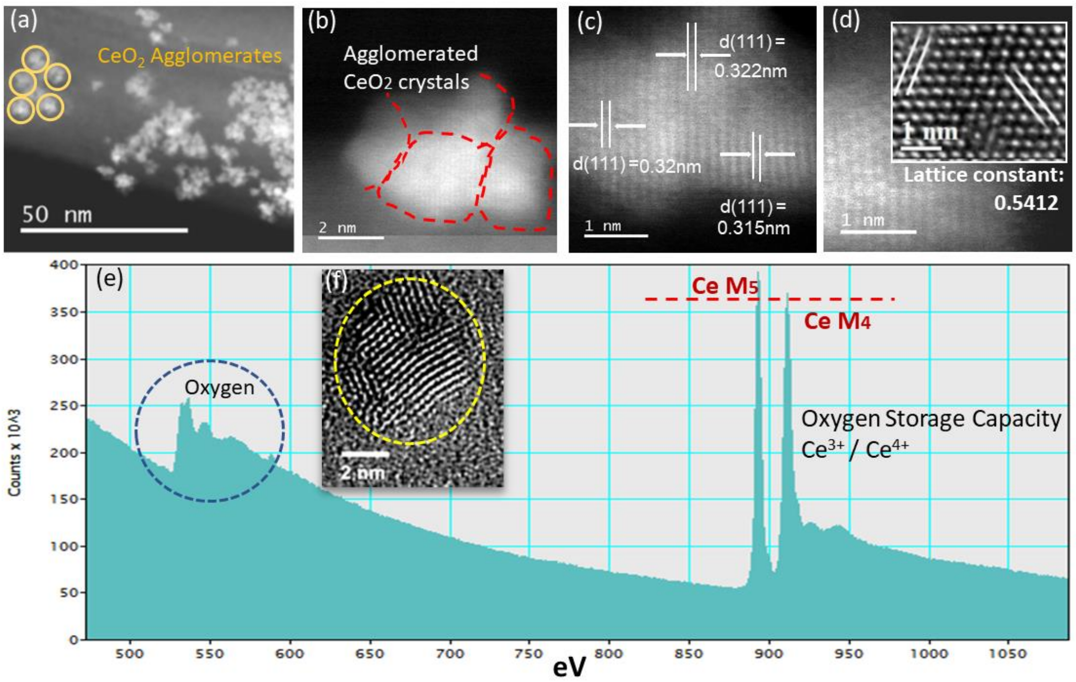

Figure S1. HRSTEM illustrates CeO_2 NPs and agglomerates (**a** – **b**) with grain boundaries marked at the intersection of individual CeO_2 NPs within an agglomerate; (**c**) shows lattice image and d-spacings for agglomerated CeO_2 NPs with (111) face; (**d**) lattice constant analyzed for CeO_2 NPs shown in b and c; EELS analysis with Ce M5 and M4 edges marked to compute the oxygen storage capacity for the crystalline 4 nm CeO_2 NP (insert in (**f**)).

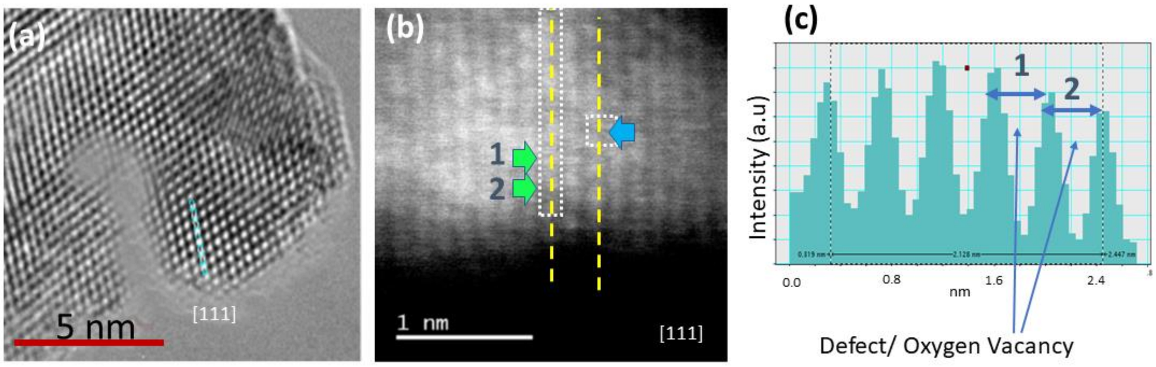

Figure S2. HRSTEM illustrates CeO_2 NPs as part of an agglomerate grain with well-formed lattice fringes seen in the interior of the CeO_2 NPs and less structure at the particle surface (**a**). (**b**) shows a higher magnification of the area with blue line marked in (**a**). (**b**) marks spots 1 and 2 in the lattice image with green arrows along the yellow trace line shown in (**c**) which measures distance for d-spacings and shows two wider spacings (defects) at spots 1 and 2 which mark potential defect sites (oxygen vacancies).

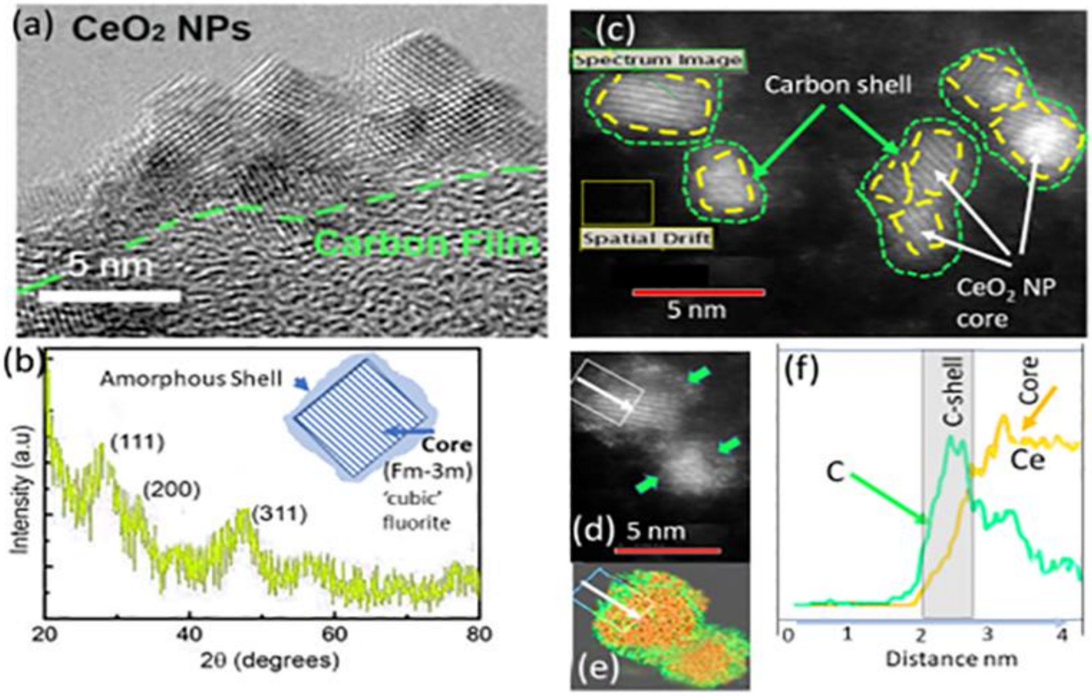

Figure S3. HRTEM image of (**a**) synthesized crystalline CeO_2 NPs (Fm-3m) adhered to carbon support film with predominantly (111) and minor (200) and (311) face and peak broadening effects due to size and surface coating (amorphous shell); (**b**) XRD analysis of CeO2 NPs; (**c**) HRSTEM of CeO_2 NPs with an amorphous outer shell (carbon shell); (**d** – **f**) Spectrum imaging with trace line from outer shell (amorphous shell) to interior of CeO_2 NPs showing Ce-rich core surrounded by a carbon shell.

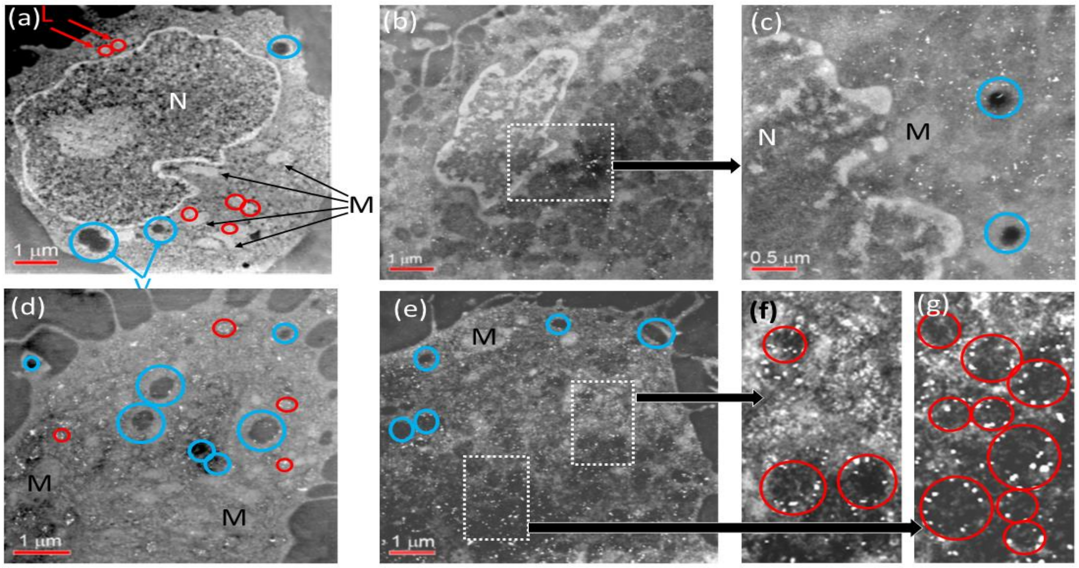

Figure S4. STEM imaging showing human blood-derived M0 cells: N=nucleus; M=macrophages; red circles=lysosomes; blue circles=vacuoles; CeO_2 NPs (white particles). M0 exposed to 10 μg/ml CeO_2 NPs for 24 h.

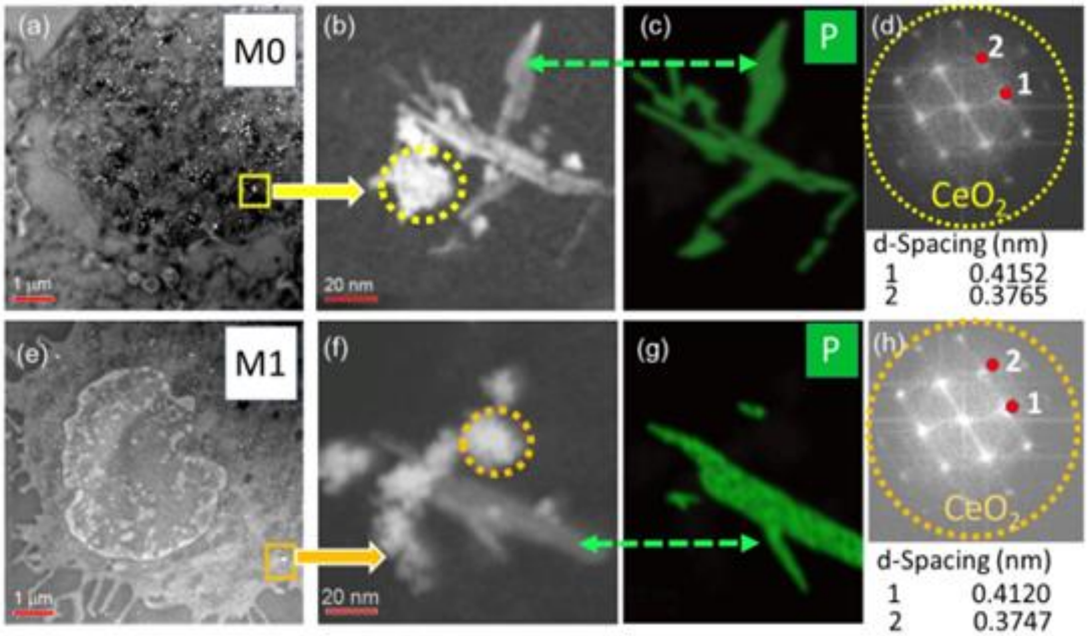

Figure S5. HRSTEM showing murine unpolarized RAW 264.7 cells. exposed to CeO_2 NPs. (**a**) White particles are translocated CeO_2 NPs; (**b**) CeO_2 NPs and $CePO_4$ needles after 24 h exposure to 10 μg/ml CeO_2 NPs; (**c**) EDS map; (**d**) electron diffraction of needle. (**e**) STEM image of murine M1 cell after exposure to CeO_2 NPs; (**f**) CeO_2 NPs and $CePO_4$ needles after 24 h exposure to 10 μg/ml CeO_2 NPs; (**g**) EDS map; (**h**) electron diffraction of needle.

**6 h**	M0	M1-like	M2-like
Untreated	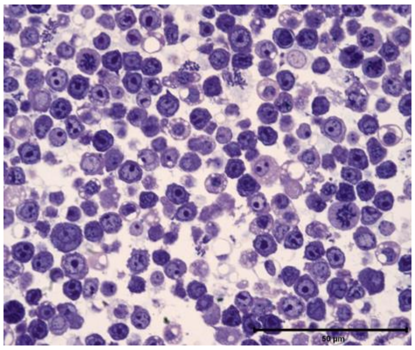	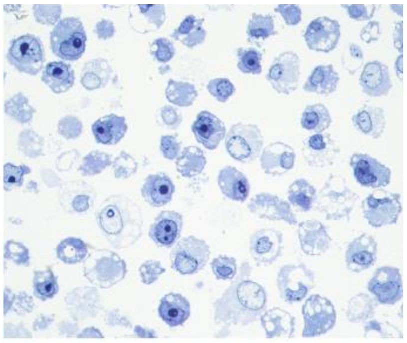	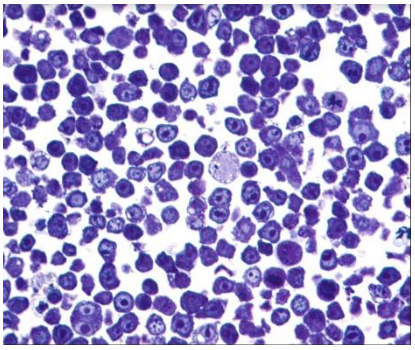
CeO_2 NP treated	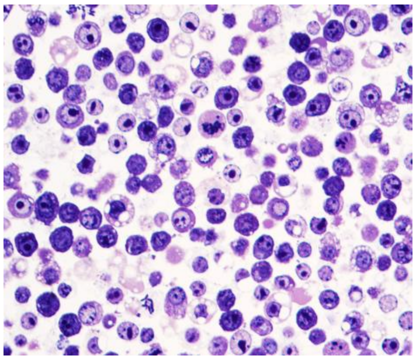	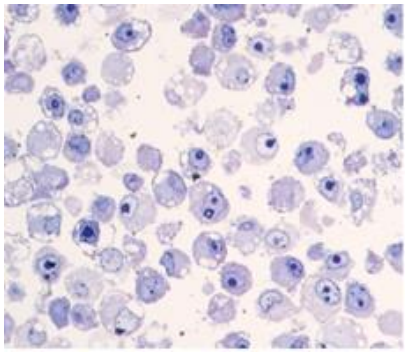	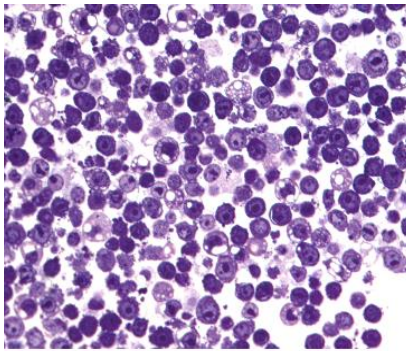
Ce ion treated	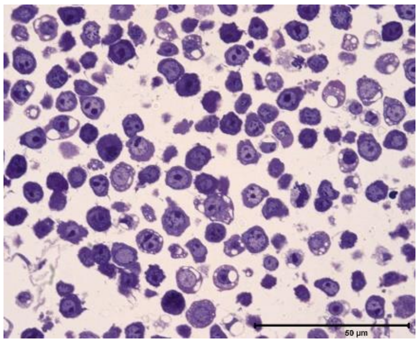	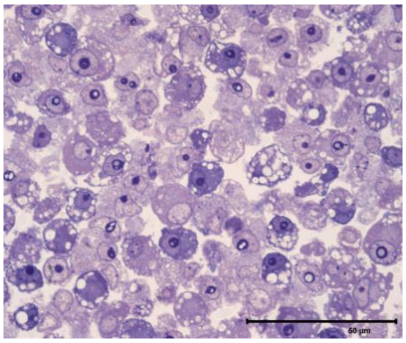	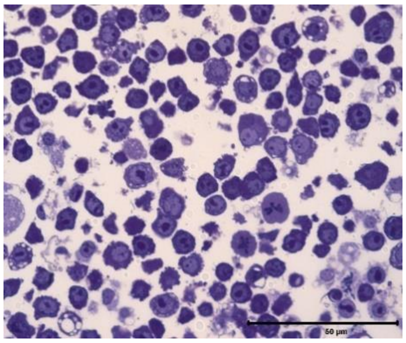

**24 h**	M0	M1-like	M2-like
Untreated	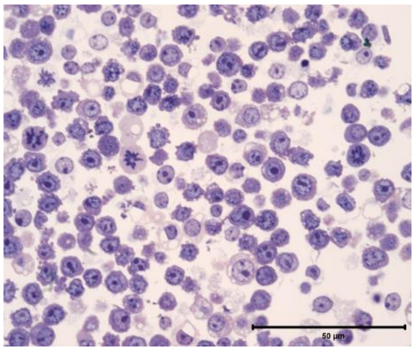	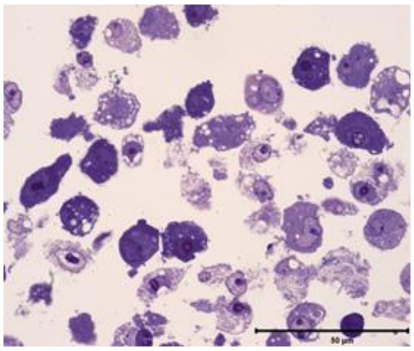	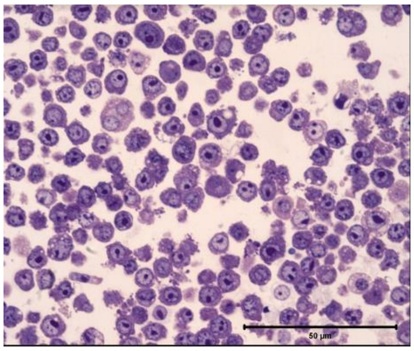
CeO_2 NP treated	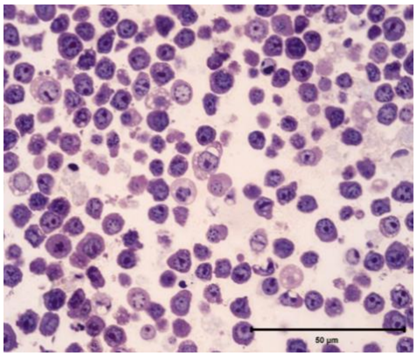	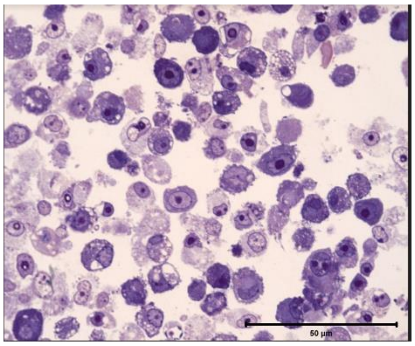	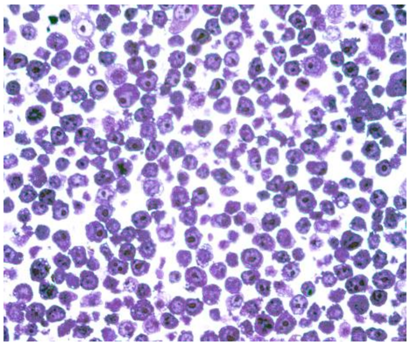
Ce ion treated	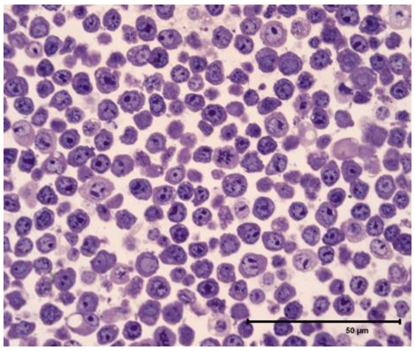	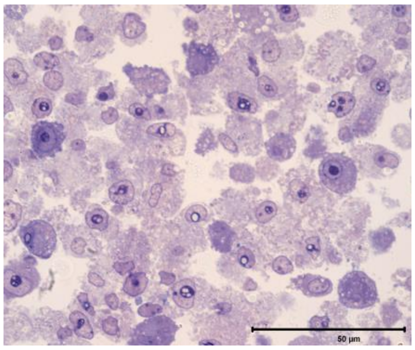	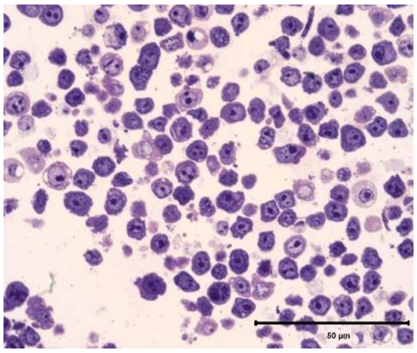

Figure S6. Light microscopic images of toluidine blue-stained M0, M1, and M2-like polarized RAW 264.7 cells. Cells that were not, or were, CeO_2 NP or Ce-ion treated for 6 or 24 h are shown. Scale bar is 50 mm in all images.

(a)

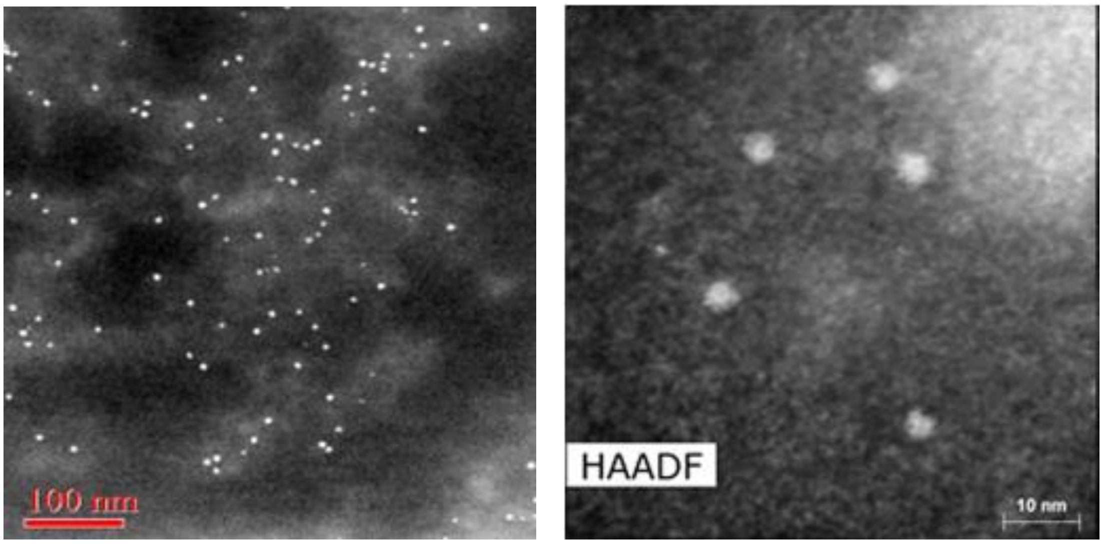

(b)

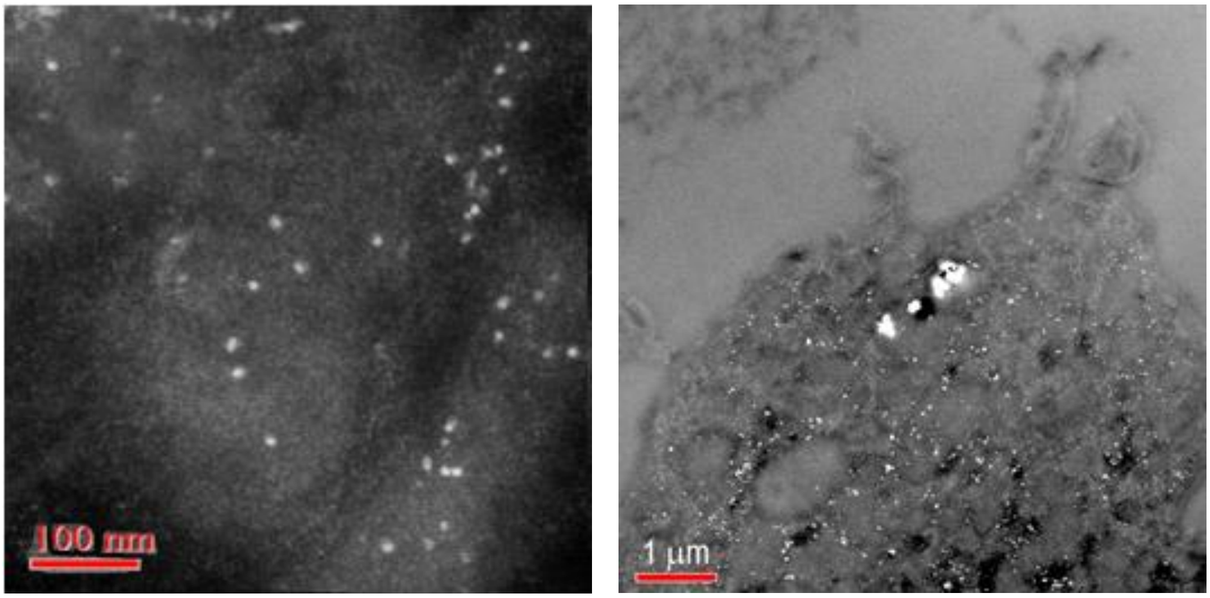

Figure S7. a: Human M0 cell with copious ferritin NPs after exposure to CeO_2 NPs. STEM-HAADF analysis shows individual ferritin NPs (white particles) at two magnifications. **b**: Human M2 cell with copious ferritin NPs after exposure to CeO_2 NPs. STEM-HAADF imaging shows individual ferritin NPs (white particles) at two magnifications.

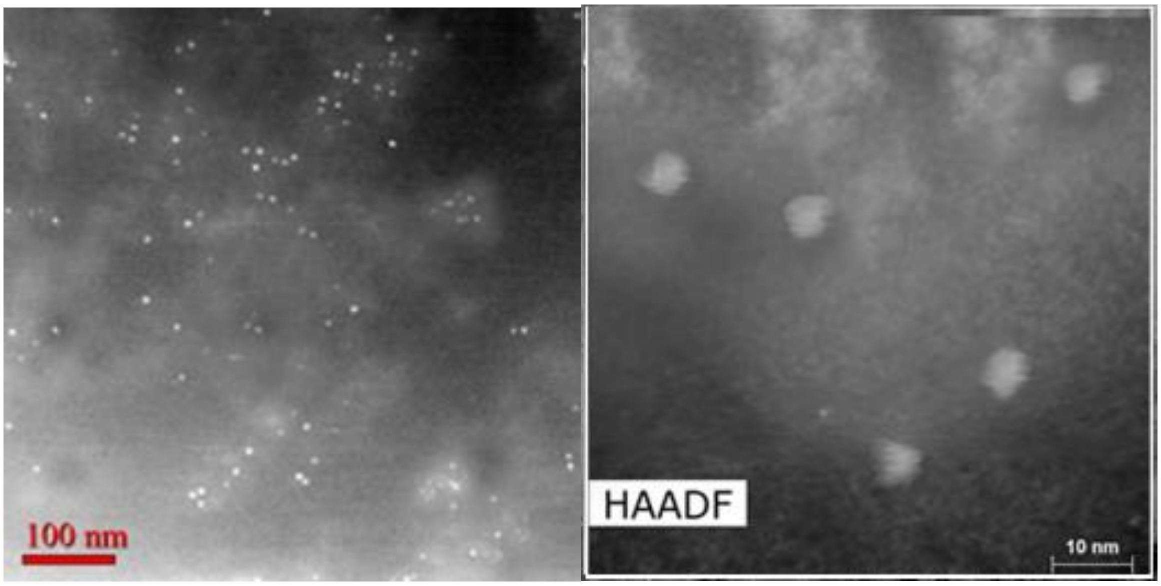

Figure S7c: Murine RAW 264.7 cell (unpolarized M0) with copious ferritin NPs that formed after exposure to Ce-ions for 6 h. STEM-HAADF imaging demonstrates ferritin NP formation is similar to that seen after CeO_2 NP exposure regarding ferritin size and dispersion.

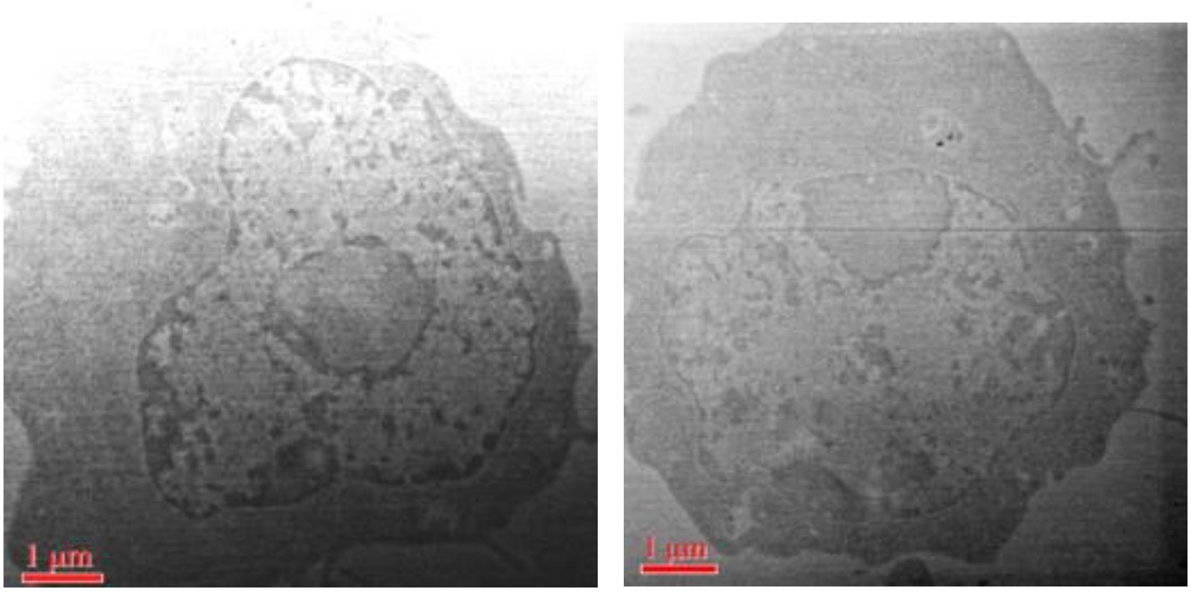

Figure S7d: HRSTEM images of untreated M1 and M2 Murine RAW 264.7 cells. Ferritin NPs were not increased in density (number of NP) in untreated M1 (left) or M2 (right) type cells.

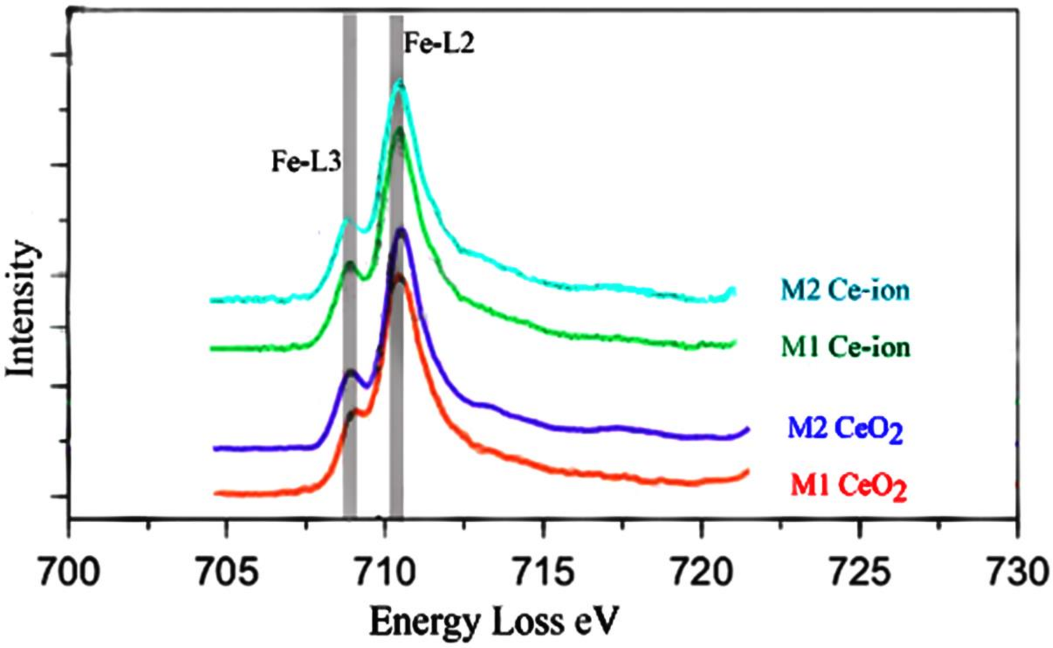

Figure S8: EELS analyses of ferritin NPs observed in M1 and M2-like cells after CeO_2 NP and Ce-ion exposure. Individual ferritin NPs are illustrated in Figures S3a –c. The Fe-L3 and Fe-L2 edges are marked with grey lines and there is ~ 1.5 eV shift between the four analyzed spectra shown.

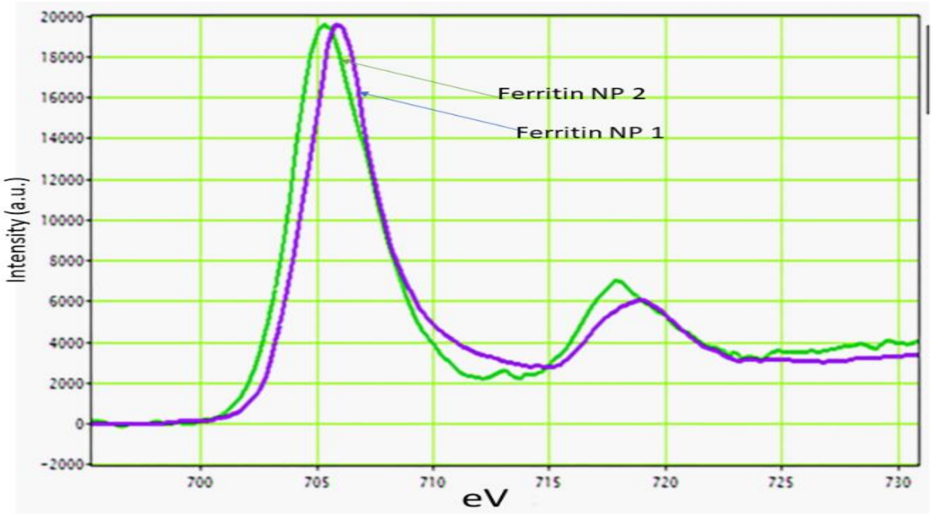

Figure S9: EELS analyses are illustrated for two distinct ferritin NP 1 and 2 that formed after CeO_2 NPs exposure to M1 and M2-type cells. The iron edges for both ferritins are separated by only 1.5 eV.
